# Anti-Diabetic Effect of *Portulaca oleracea* L. Polysaccharideandits Mechanism in Diabetic Rats

**DOI:** 10.3390/ijms17081201

**Published:** 2016-07-25

**Authors:** Yu Bai, Xueli Zang, Jinshu Ma, Guangyu Xu

**Affiliations:** 1 Pharmaceutical College, Jilin Medical University, Jilin 132011, China; 2 Department of Pharmacy, Changchun Medical College, Changchun 130033, China; zangxueli1980@163.com; 3 Department of Pathology, China-Japan Union Hospital of Jilin University, Changchun 130033, China; jjsmadina@126.com; 4 College of Pharmacy, Beihua University, Jilin 132013, China; xuguangyu2005@163.com

**Keywords:** purslane, polysaccharide, anti-diabetic

## Abstract

Diabetes mellitus (DM) is a metabolic syndrome caused by multiple genetic and environmental factors. Traditional Chinese medicine preparations have shown a comprehensive and function-regulating characteristic. Purslane (*Portulaca oleracea* L.) is an annual succulent herb. Currently, there have been some related reports on the treatment of diabetes with purslane. The current study was designed to separate and purify the polysaccharide, a systematic study of its physical and chemical properties, antioxidant activity, and anti-diabetic mechanism, in order to provide a theoretical basis for the development of drugs of purslane. A crude water soluble polysaccharide extracted from purslane was named CPOP (crude *Portulaca oleracea* L. polysaccharide). Effects of CPOP on bodyweight, glucose tolerance test (GTT), fasting blood glucose (FBG), fasting serum insulin (FINS), insulin sensitivity index (ISI), interleukin-6 (IL-6), tumor necrosis factor-α (TNF-α), methane dicarboxylic aldehyde (MDA), and superoxygen dehydrogenises (SOD) were investigated. The results indicate that the oral administration of CPOP could significantly increase the body weight and significantly improve the glucose tolerance in diabetic rats. Meanwhile, CPOP could significantly reduce the FBG level, and elevate the FINS level and ISI value in diabetic rats. In addition, CPOP could significantly reduce TNF-α and IL-6 levels in diabetic rats; CPOP could also reduce MDA and SOD activities in the liver tissue of diabetic rats. These results suggest that the anti-diabetic effect of CPOP may be associated with its antioxidant and anti-inflammatory effects.

## 1. Introduction

Type II diabetes mellitus, an endocrine and metabolic disease caused by the combined effects of polygenic and environmental factors, accounts for more than 90 percent of all diabetic patients [1]. The condition is characterized by a relative lack of insulin secretion and/or insulin resistance [2]. According to the World Health Organization, the number of diabetic patients across the world has reached 200 million, and is expected to exceed 336 million by year 2030 [3]. Oral hypoglycemic therapy with drugs, such as biguanides [4], thiazolidinediones (TZDs) [5], and glucosidase [6] inhibitors, is the primary therapeutic modality for type II diabetes. Despite their efficacy in maintaining glycemic control, oral hypoglycemic agents may not prevent the long-term complications of diabetes, such as nephropathy [7], and cardiovascular [8] disorders. Further, long-term use of these drugs is often associated with serious side effects, such as gastrointestinal disorders [9] associated with use of acarbose; granulocytopenia and hypoglycemia with glibenclamide; and lactic acidosis [10] associated with metformin therapy.

In traditional Chinese medicine, diabetes is referred to as “Xiaokezheng” or “Xiaobing” and an abundant clinical experience of its treatment has accumulated over the years [11].

A total of 187 different traditional Chinese remedies for diabetes have been documented in the Compendium of Materia Medica, a famous Chinese medicine work. As compared to the modern chemical drugs, the traditional Chinese medicine is known to modulate physiological regulation that effectively prevents or delays the multi-systemic long-term complications of diabetes in addition to lowering blood sugar levels [12,13,14,15,16]. In addition, Chinese medicine typically has a lower propensity for severe toxicity and adverse reactions. Therefore, research into Chinese herbal remedies for diabetes has evoked considerable interest.

Purslane is an annual succulent herb with succulent leaves that may grow prostrate or erect depending on light availability [17], which is distributed all over the world, and grows well in diverse geographical environments [18,19]. Purslane belongs to family Portulacaceae and is classified as a C4 plant, which is listed as one of the most useful medicinal plants and named “Global Panacea” by the World Health Organization [20]. It is a traditional Chinese herb now widely distributed throughout the world. The active ingredients include polysaccharides [21], fatty acids [22], flavonoids [22], coumarin [22], and alkaloids [23]. It is rich in antioxidant vitamins and omega-3 fatty acids [20] and can be used as a vegetable as well as for various curative purposes in health care, especially in preventing some cardiovascular diseases and maintaining a healthy immune system [24]. It is known to have antibacterial, anti-inflammatory, and antioxidant properties, and is known to regulate lipid and sugar metabolism in the body. The aqueous extract of *Portulaca oleracea* also prevents diabetic vascular inflammation, hyperglycemia, and diabetic endothelial dysfunction in type II diabetic *db*/*db* mice, suggesting its protective role against diabetes and related vascular complications [25]. The crude polysaccharide extract of this plant also lowers blood glucose and modulates the metabolism of blood lipids and glucose in alloxan-induced diabetic mice [26], whilst decreasing the levels of total cholesterol, triglycerides, and fasting blood glucose in type II diabetic mice [27].

Use of purslane polysaccharides for treatment of diabetes has not been rigorously evaluated. In this study, purslane polysaccharides was separated and purified, and a systematic study of its physical and chemical properties, antioxidant activity, as well as its anti-diabetic mechanism systematically examined. The objective is to provide a theoretical basis for the therapeutic use of purslane polysaccharides in the treatment of diabetes.

## 2. Results and Discussion

### 2.1. Polysaccharide Characterization

**Figure 1:**
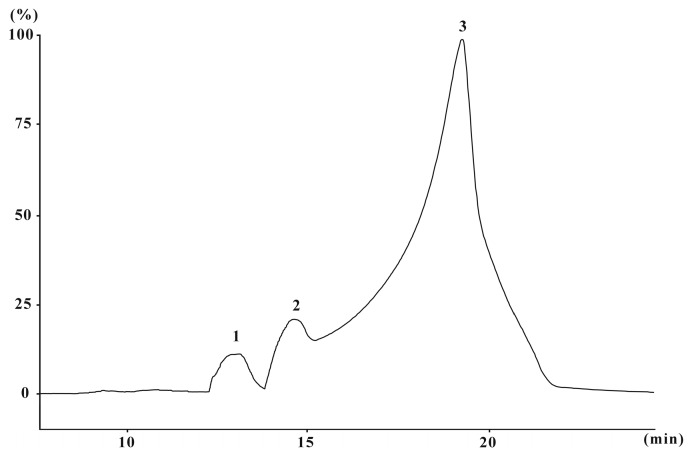
High-performance size-exclusion chromatography (HPSEC) chromatogram of CPOP (crude *Portulaca oleracea* L. polysaccharide). 1: 9.3 × 10^4^ Da, 2: 11.9 × 10^3^ Da, 3: 7.3 × 10^3^ Da.

**Figure 2:**
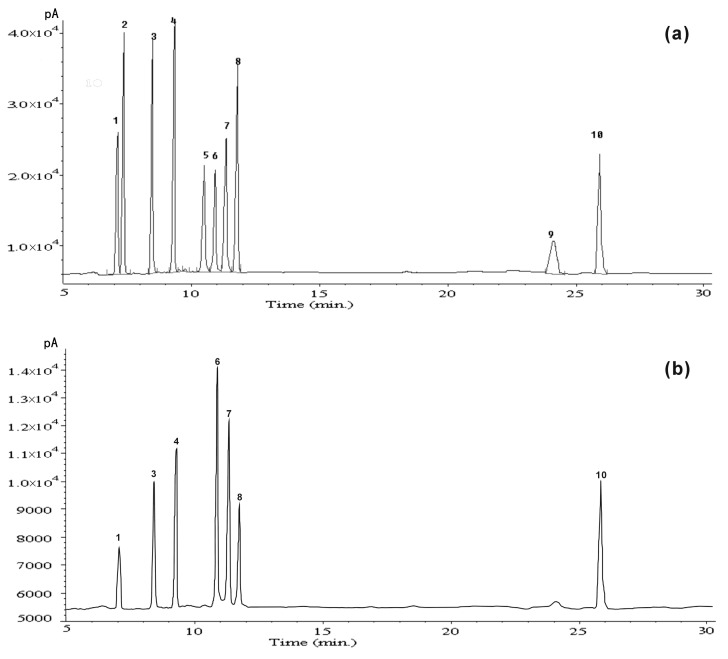
(**a**) Gas chromatography (GC) chromatogram of mixed standard monosaccharides derivatives; (**b**) GC chromatogram of hydrogen derivative from CPOP. Peak identification: 1: rhamnose, 2: fructose, 3: arabinose, 4: xylose, 5: mannose, 6: galactose, 7: glucose, 8: myo-inositol, 9: glucuronic acid, and 10: galacturonic acid.

The yield of CPOP (crude *Portulaca oleracea* L. polysaccharide) approximated 9.6% of the dry weight of raw material with 48.3% total carbohydrates (by phenol-sulfuric acid), 10.3% proteins, and 40.5% uronic acid. The molecular weight of CPOP was determined and calculated by high-performance size-exclusion chromatography. CPOP showed one main molecular weight distribution (7.3 × 10^3^ Da) and two minor molecular weight distributions (11.9 × 10^3^ and 9.3 × 10^4^ Da) (Figure 1). The neutral monosaccharide composition was assessed by gas chromatography, which showed the presence of rhamnose, arabinose, xylose, mannose, glucose, and galactose in the ratio of 1:1.1:1.3:1.9:2.4:3.4:1 (Figure 2).

### 2.2. General Condition and Body Weight of Diabetic Rats

The mental status of rats in the normal control (NC) group appeared normal and they showed good response and movement. Their fur was shiny and their body weights increased steadily. After the intraperitoneal injection of streptozotocin (STZ), rats in the model control group (MC) showed a tendency for increased intake of food and water, had an increased urine output and thin and soft stools. Further, these rats showed progressive loss of body weight, appeared depressed with sluggish response, lethargic movement, and untarnished fur. After oral administration of CPOP and glyburide, the mental state of diabetic rats appeared to improve significantly. The body weight of rats in the 200-CPOP and 400-CPOP groups increased significantly (*p* < 0.05 or *p* < 0.01) (Table 1).

### 2.3. Effects of CPOP (Crude Portulaca oleracea L. Polysaccharide) on Glucose Tolerance in Diabetic Rats

The development of type II diabetes mellitus progresses from a state of normal glucose tolerance (NGT) to impaired glucose tolerance (IGT) and, finally, diabetes [28]. Thus, intervention during the state of IGTs appears to be a key to the prevention and treatment of type II diabetes mellitus. As shown in Table 2, after the overnight fast, blood sugar levels of rats that had been injected 20% sterile glucose solution in NC, MC, CPOP, and glyburide groups, reached their peak values at the first 30 min and, thereafter, gradually decreased; at the 120th min post-injection, the blood sugar levels in the NC group were restored to their normal basal level, while those in the MC, CPOP, and glyburide groups remained at a high level, indicating reduced glucose tolerance in rats in those groups.

Compared with that in MC group, the fall in blood sugar levels in type II diabetic rats that had been injected with the glucose solution in the CPOP groups were significantly accelerated, suggesting a potential improvement in glucose tolerance induced by CPOP. Compared with those at the first 30 min, decreased rates of blood glucose levels at the 60th and 120th min were, respectively, 13.01% and 32.7% in rats in the MC group, while those in 100-CPOP, 200-CPOP, 400-CPOP, and glyburide groups at the 60th min were 21.67%, 27.10%, 32.01%, and 33.12%, respectively; those at the 120th min were 41.48%, 44.35%, 49.54%, and 52.39%, respectively.

### 2.4. Effects of CPOP on Fasting Blood Glucose (FBG), Fasting Serum Insulin (FINS) and Insulin Sensitivity Index (ISI)

**Figure 3:**
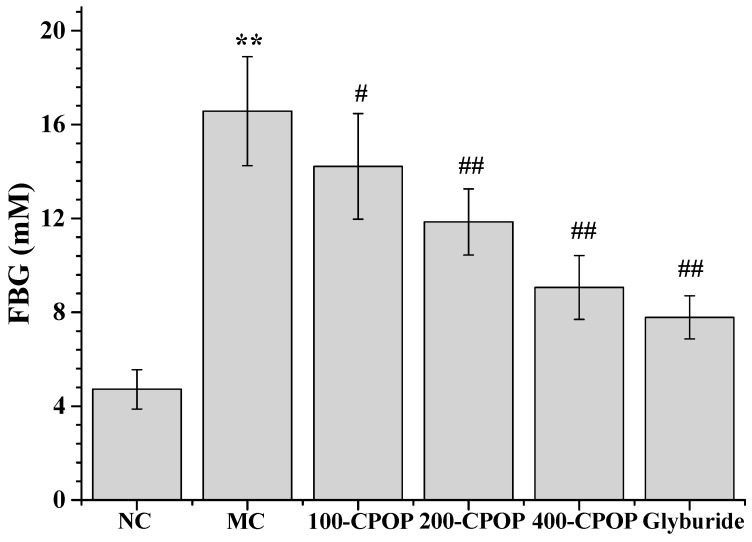
Effect of CPOP on Fasting Blood Glucose (FBG) in Streptozotocin (STZ)-induced diabetic rats. ** *p* < 0.01 vs. NC group (*N* = 8); ^#^ *p* < 0.05, ^##^ *p* < 0.01 vs. MC group (*N* = 8). NC is the normal control group; MC is the model control group; 100-CPOP is the 100 mg/kg per body weight CPOP group; 200-CPOP is the 200 mg/kg per body weight CPOP group; 400-CPOP is the 400 mg/kg per body weight CPOP group.

**Figure 4:**
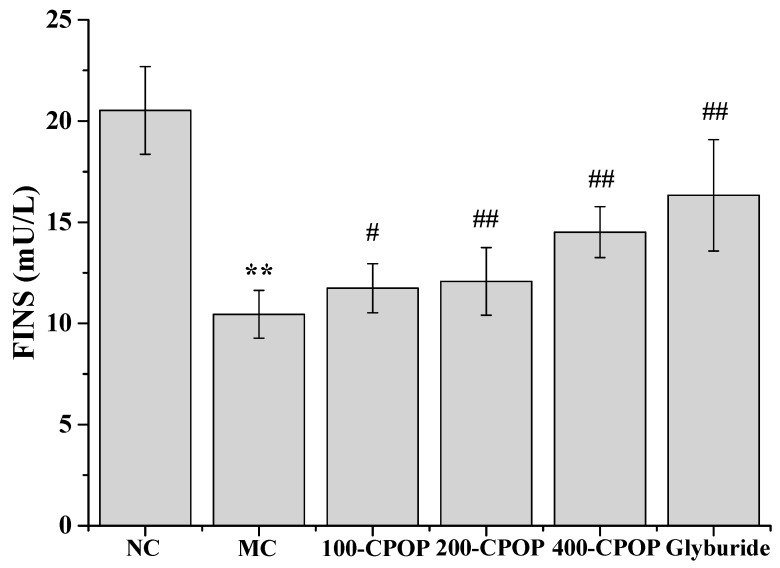
Effect of CPOP on Fasting Serum Insulin (FINS) in STZ-induced diabetic rats. ** *p* < 0.01 vs. NC group (*N* = 8); ^#^ *p* < 0.05, ^##^ *p* < 0.01 vs. MC group (*N* = 8). NC is the normal control group; MC is the model control group; 100-CPOP is the 100 mg/kg per body weight CPOP group; 200-CPOP is the 200 mg/kg per body weight CPOP group; 400-CPOP is the 400 mg/kg per body weight CPOP group.

**Figure 5:**
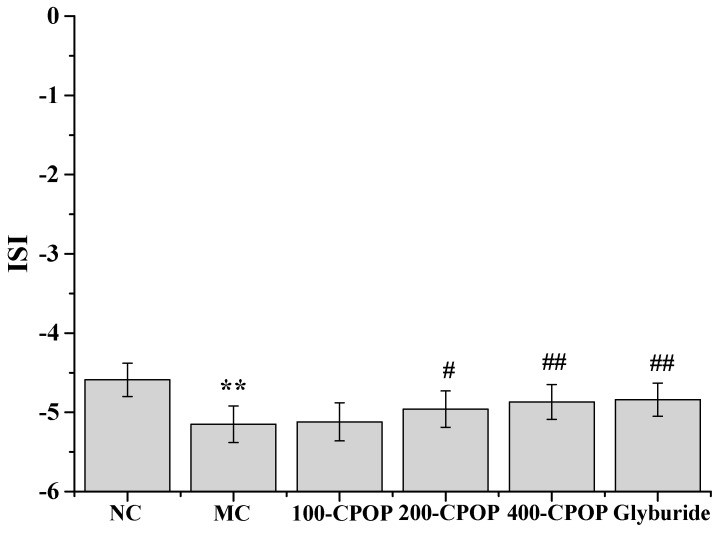
Effect of CPOP on Insulin Sensitivity Index (ISI) in STZ-induced diabetic rats. ** *p* < 0.01 vs. NC group (*N* = 8); ^#^ *p* < 0.05, ^##^ *p* < 0.01 vs. MC group (*N* = 8). NC is the normal control group; MC is the model control group; 100-CPOP is the 100 mg/kg per body weight CPOP group; 200-CPOP is the 200 mg/kg per body weight CPOP group; 400-CPOP is the 400 mg/kg per body weight CPOP group.

As illustrated in Figure 3 and Figure 4, compared with those in the NC group, Fasting Blood Glucose (FBG) values were significantly higher and Fasting Serum Insulin (FINS) levels significantly lower in the MC group (*p* < 0.01). Compared with those in the MC group, FBG levels were significantly lower, while FINS levels significantly higher in rats in CPOP groups (*p* < 0.05 or *p* < 0.01). As shown in Figure 5, compared with those in the MC group, Insulin Sensitivity Index (ISI) values were higher in the 100-CPOP, 200-CPOP and 400-CPOP groups, of which those in the 200-CPOP and 400-CPOP groups increased significantly (*p* < 0.05 or *p* < 0.01).

### 2.5. Effects of CPOP on Tumor Necrosis Factor-α (TNF-α) and Interleukin-6 (IL-6) Levels in Diabetic Rats

**Figure 6:**
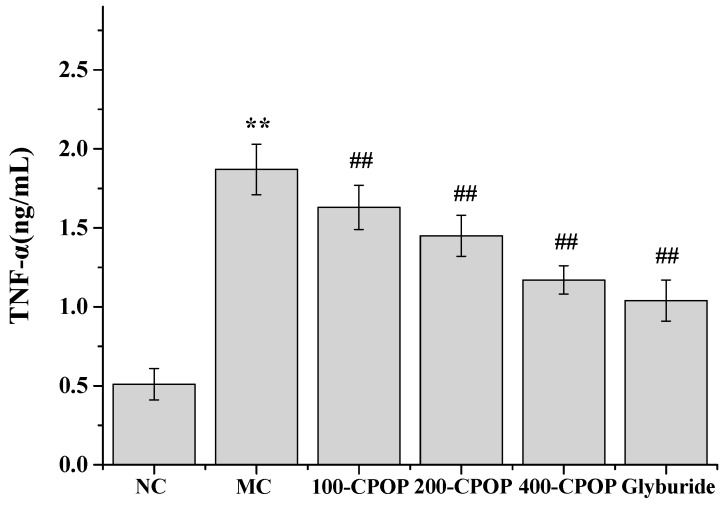
Effect of CPOP on tumor necrosis factor-α (TNF-α) levels in STZ-induced diabetic rats. ** *p* < 0.01 vs. NC group (*N* = 8); ^##^ *p* < 0.01 vs. MC group (*N* = 8). NC is the normal control group; MC is the model control group; 100-CPOP is the 100 mg/kg per body weight CPOP group; 200-CPOP is the 200 mg/kg per body weight CPOP group; 400-CPOP is the 400 mg/kg per body weight CPOP group.

**Figure 7:**
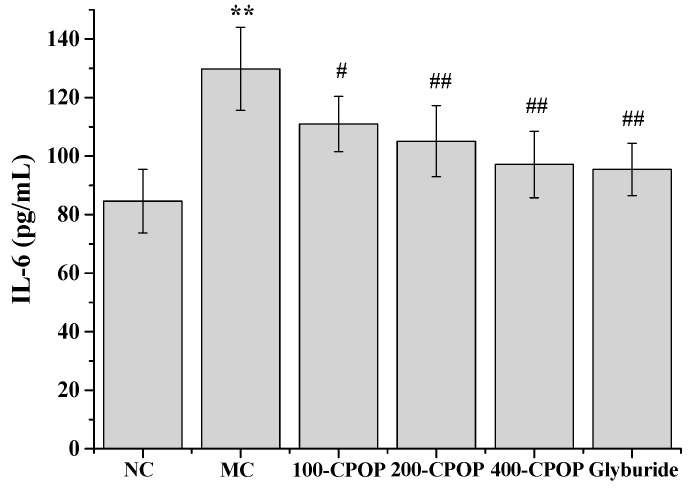
Effect of CPOP on interleukin-6 (IL-6) levels in STZ-induced diabetic rats. ** *p* < 0.01 vs. NC group (*N* = 8); ^#^ *p* < 0.05, ^##^ *p* < 0.01 vs. MC group (*N* = 8). NC is the normal control group; MC is the model control group; 100-CPOP is the 100 mg/kg per body weight CPOP group; 200-CPOP is the 200 mg/kg per body weight CPOP group; 400-CPOP is the 400 mg/kg per body weight CPOP group.

In recent years, a large number of studies have implicated cytokine-mediated inflammation in the pathogenesis of type II diabetes mellitus [29,30]. Several inflammatory cytokines, such as tumor necrosis factor-α (TNF-α) and interleukin-6 (IL-6), have been implicated in the causation of insulin resistance and appear to confer an increased risk of micro vascular complications in type II diabetes mellitus [31,32]. The TNF-α and IL-6 levels were significantly higher in the MC group, as compared to those in the NC group (*p* < 0.01). Further, as compared with those in MC group, TNF-α and IL-6 levels were significantly lower in the CPOP-treated diabetic rats on 28th day after oral administration of 100, 200, and 400 mg/kg CPOP (*p* < 0.05 or *p* < 0.01) (Figure 6 and Figure 7).

### 2.6. Effects of CPOP on Methane Dicarboxylic Aldehyde (MDA) Contents and Superoxygen Dehydrogenises (SOD) Activities in Diabetic Rats

Although the molecular mechanisms of diabetes and its associated complications remain unclear, an increasing body of evidence appears to implicate the reactive oxygen species (ROS) in the pathogenesis of diabetes and its complications [33]. The strong correlation between diabetes and oxidative stress is well documented [34]. Superoxide dismutase, an endogenous antioxidase, serves to ameliorate the toxic effects of the oxygen radicals. Methane dicarboxylic aldehyde (MDA), an important lipid peroxide, is a sensitive index of metabolic levels of free radicals.

**Figure 8:**
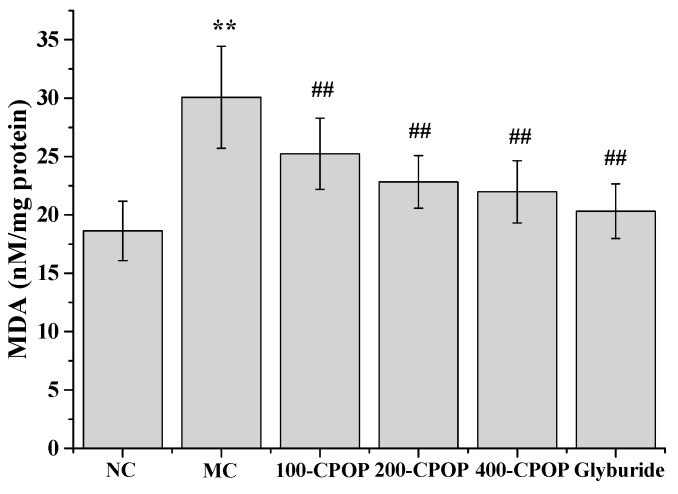
Effect of CPOP on methane dicarboxylic aldehyde (MDA) contents in liver tissues of STZ-induced diabetic rats. ** *p* < 0.01 vs. NC group (*N* = 8); ^##^ *p* < 0.01 vs. MC group (*N* = 8). NC is the normal control group; MC is the model control group; 100-CPOP is the 100 mg/kg per body weight CPOP group; 200-CPOP is the 200 mg/kg per body weight CPOP group; 400-CPOP is the 400 mg/kg per body weight CPOP group.

**Figure 9:**
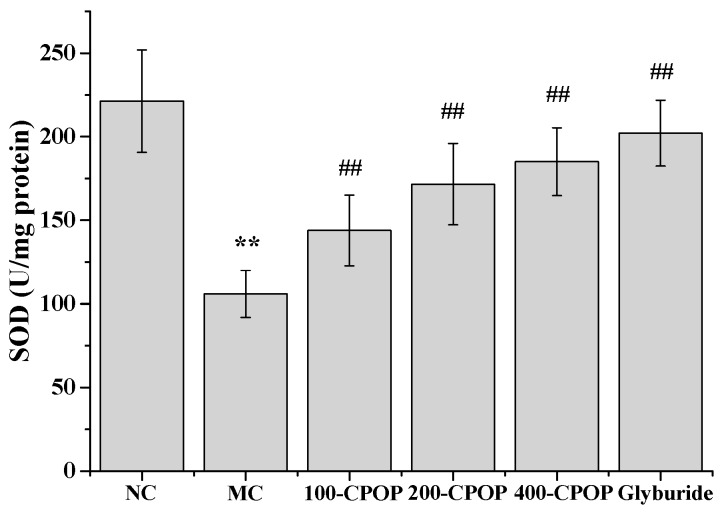
Effects of CPOP on superoxygen dehydrogenises (SOD) activities in liver tissues of STZ-induced diabetic rats. ** *p* < 0.01 vs. NC group (*N* = 8); ^##^ *p* < 0.01 vs. MC group (*N* = 8). NC is the normal control group; MC is the model control group; 100-CPOP is the 100 mg/kg per body weight CPOP group; 200-CPOP is the 200 mg/kg per body weight CPOP group; 400-CPOP is the 400 mg/kg per body weight CPOP group.

The MDA content in rat liver tissue was significantly higher, while superoxygen dehydrogenises (SOD) activity was significantly lower in the MC group, as compared to those in the NC group (*p* < 0.01).On the 28th day after the intragastric administration of 100, 200, and 400 mg/kg CPOP, MDA content was lower, while the SOD activity was higher in the 100-CPOP, 200-CPOP, and 400-CPOP groups as compared to those in the MC group. Further, the difference in MDA content and SOD activity between the MC and CPOP groups was found to be statistically significant (*p* < 0.01) (Figure 8 and Figure 9).

### 2.7. Effects of CPOP on Protein Tyrosine Phosphatase 1B (PTP1B) Activities in Diabetic Rats

Protein tyrosine phosphatase 1B (PTP1B), an ideal therapeutic target for type II diabetes, has become the intense pharmaceutical interest for treating type II diabetes over the past decade [35]. Considering that PTP1B directly dephosphorylates insulin receptors and the receptor substrate, thereby negatively regulating the insulin signaling pathway, it may also have a direct effect on its downstream pathways by affecting the activity or expression of insulin signal transduction molecules to inhibit insulin signal transduction, leading to insulin resistance [36,37]. We expected CPOP to be useful in the treatment of type II diabetes [38]. The active ingredient having PTP1B inhibition activity has been extracted from a variety of natural herbs and plants [39].

**Figure 10:**
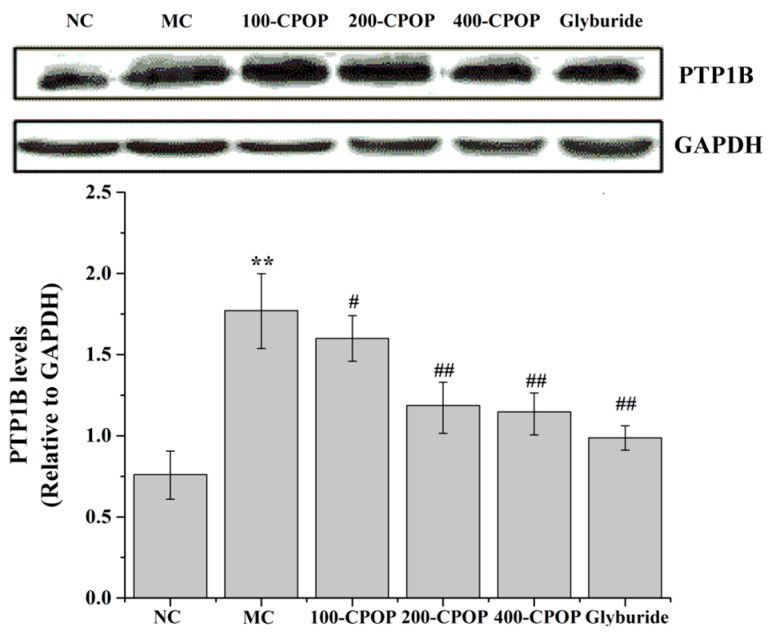
Effects of CPOP on protein tyrosine phosphatase 1B (PTP1B) levels in liver tissues of STZ-induced diabetic rats. ** *p* < 0.01 vs. NC group (*N* = 8); ^#^ *p* < 0.05, ^##^ *p* < 0.01 vs. MC group (*N* = 8). NC is the normal control group; MC is the model control group; 100-CPOP is the 100 mg/kg per body weight CPOP group; 200-CPOP is the 200 mg/kg per body weight CPOP group; 400-CPOP is the 400 mg/kg per body weight CPOP group. GAPDH: glyceraldehyde-3-phosphate dehydrogenase.

The results of this study found that the model group PTP1B levels were significantly higher (*p* < 0.01); compared with the model group, PTP1B content which of diabetic rats in the 100-CPOP, 200-CPOP, and 400-CPOP after 28 days decreased in a dose-dependent manner (*p* < 0.05 or *p* < 0.01) (Figure 10). These studies indicate that PTP1B inhibitors may be promising candidates for novel anti-diabetic drug development.

## 3. Materials and Methods

### 3.1. Materials and Chemicals

Machixian (*Portulaca oleracea* L.), was purchased from Jilin Farmer’s Market and identified by Zhang Lihua at the College of Pharmacy, Beihua University, Jilin, China; Streptozotocin (STZ) was acquired from Sigma-Aldrich, Shanghai, China (80,082,038); glibenclamide was acquired from Zhejiang Nanyang Pharmaceutical Co., Ltd. (Hangzhou, China) (20121101); fasting glucose/fasting blood glucose (FBG) test kits were acquired from Sichuan Mike Technology Shares Limited Liability Company (Chengdu, China) (1,301,014); fasting insulin assay kit/fasting serum insulin (FINS) were acquired from Beijing Northern Institute of Biotechnology (Beijing, China) (130,219); tumor necrosis factor-α (TNF-α) detection kit was acquired from Beijing Northern Institute of Biotechnology (120,518); interleukin-6 (IL-6) detection kit was acquired from Wuhan Boster Biological Engineering Co., Ltd. (Wuhan, China) (20120316); Malondialdehyde (MDA) detection kit was acquired from Nanjing Jiancheng Institute of Biotechnology (Nanjing, China) (201,211,108); superoxide dismutase (SOD) detection kit was acquired from Nanjing Jiancheng Institute of Biotechnology (20,121,214). Trifluoroacetic acid (TFA), T-series dextrans (T-2000, T-70, T-40, T-20, and T-10) and the standard monosaccharides (rhamnose, fucose, arabinose, xylose, mannose, galactose, glucose, galacturonic acid) were acquired from Sigma Chemical Co. (St. Louis, MO, USA). All the other chemical reagents were of analytical grade.

### 3.2. Extraction of Polysaccharide of Portulaca oleracea L.

The residual root and impurities of fresh *Portulace oleracea* L. (1000 g) were removed and the herb washed, dried at 60 °C, pulverized, and extracted twice with ethanol. The residues were extracted with hot water at 80 °C (1:20, *w*/*v*) three times, at 3 h each. The extracted solution was mixed and concentrated, and the associated proteins removed using the Sevag method [40]. The solution was concentrated and precipitated with four volumes of 95% (*v*/*v*) ethanol at 4 °C for 24 h in order to isolate the polysaccharide. The precipitate was washed with absolute ethanol, acetone and ether, respectively. Finally, the precipitate was suspended in water and lyophilized to yield the crude polysaccharide, referred to as crude *Portulaca oleracea* L. polysaccharide (CPOP).

### 3.3. Characterization of CPOP

The total carbohydrate content in the CPOP fraction was determined by the phenol-sulfuric acid method and the glucose was taken as the standard [41]. The protein content was quantified according to the folin-phenol method, using Bovine Serum Albumin (BSA) as standard [42]. Total uronic acid contents were measured by m-hydroxydiphenyl method using galacturonic acid as the standard [43].

### 3.4. Monosaccharide Composition Analysis

The identification and quantification of the monosaccharides in CPOP was achieved by gas chromatography (GC). Polysaccharides (10 mg) were hydrolyzed with 2 M TFA at 100 °C for 2 h, followed by drying, reduction with NaBH_4_, and acetylation with Ac_2_O-NaOAc at 120 °C for 1 h [44]. The Ac_2_O was denatured with ice water, and the resulting alditol acetate extracted with chloroform and examined by GC. The following neutral monosaccharides were used as references: rhamnose, fucose, arabinose, xylose, mannose, galactose, and glucose. GC was performed on a Varian model 3300 instrument (Varian, Palo Alto, CA, USA) equipped with a DB-225 capillary column (Agilent, Beijing, China, 30 m × 0.25 mm inside diameter (i.d.)) and detected with a flame ionization detector (Agilent, 260 °C).The column temperature was increased from 150 to 200 °C at the rate of 4 °C/min and then held for 5 min. The quantification was carried out from the peaks area, using response factors from standard monosaccharide.

### 3.5. Molecular Weight Determination

The molecular weight of CPOP was determined by high-performance size-exclusion chromatography (HPSEC) performed on a SHIMADZU HPLC system (SHIMADZU, Suzhou, China) equipped with one TSK-G3000PWXL column (TOSOH, Tokyo, Japan, 7.8 mm i.d. × 30.0 cm) and a SHIMADZU RID-10A detector. The mobile phase was 0.7% Na_2_SO_4_ and the flow rate was 0.5 mL/min. The sample was dissolved in the mobile phase and centrifuged (10,000 rpm; 35 min), and 20 µL of supernatant injected on each run. Dextran standards with different molecular weights (T-2000, T-70, T-40, T-20, and T-10) were used to calibrate the column and establish a standard curve.

### 3.6. Animals

Special pathogen freeanimal (SPF) Sprague Dawley rats, each weighing 200 ± 20 g, were purchased from the Experimental Center at the Jilin University, Jilin, China. All experimental procedures were approved by the Animal Ethics Committee at the Beihua University, Jilin, China. The rats were housed in polypropylene cages under controlled conditions (22 ± 0.5 °C and a 12:12-hour light/dark cycle) and on a standard laboratory feed with free access to both food and water. The rats were acclimatized for one week before the start of the experiments.

### 3.7. Establishment of Rat Diabetic Model

The rats were kept in an environment with a relative humidity of 40%–70% and 22 ± 1 °C temperature with free access to both water and food. After a 12 h fasting period, rats were administered 60 mg/kg newly-prepared STZ solution (dissolved in 0.1 M citrate buffer, pH 4.2) via intra-peritoneal injection. Those in the normal control group (NC) were injected the same volume of citrate buffer. At 72 h after STZ injection, blood samples were collected by tail bleeding. The fasting blood glucose levels were measured. Rats with fasting blood glucose levels of ≥11.1 mM were selected as the rat diabetic model.

### 3.8. Grouping and Administration

The diabetic rats were randomly divided into control group, model group (MC), 100-CPOP group (100 mg/kg per body weight), 200-CPOP group (200 mg/kg per body weight), 400-CPOP group (400 mg/kg per body weight), and glyburide group (25 mg/kg per body weight). On day 2 after the modeling, oral administration of the above described drugs was started once a day for 28 days. Rats in the 100-CPOP, 200-CPOP, 400-CPOP, and glyburide groups were administered the corresponding agents, and those in the control group (NC) and the model group (MC) were administered identical volumes of saline. Pre- and post- treatment general condition of the rats and changes in body weight were documented.

### 3.9. Glucose Tolerance Test

On day 21 of treatment, the rats were fasted for 12 h, and injected 20% sterile glucose solution (2 g/kg) intraperitoneally. The blood was obtained by tail bleeding and the serum separated. Blood glucose levels at 0, 30, 60, and 120 min after glucose injection were measured with the glucose oxidase method.

### 3.10. Detection and Calculation of FBG, FINS and ISI

On day 28 of treatment, rats were anesthetized by intraperitoneal injection of 100 mg/kg urethane and blood specimens obtained from the aorta abdominalis. Specimens were centrifuged (3000 rpm, 10 min) to obtain the serum, which was kept in tubes and frozen for use. FBG levels were measured by the glucose oxidase method, FINS levels were determined by radioimmunoassay, and ISI values were calculated based on the following formula:

ISI = ln (FBG × FINS)^−1^

### 3.11. Detection of Serum Interleukin-6 and Tumor Necrosis Factor-α Levels

Enzyme-linked immuneosorbent assay (ELISA) for serum levels of IL-6 and TNF-α levels were performed as per the instructions accompanying the ELISA kits.

### 3.12. Methanedicarboxylic Aldehyde Content and Super Oxygen Dehydrogenase Activity in Liver

On day 28 after administration, the rats were sacrificed after obtaining blood samples via the aorta abdominalis. The liver tissue was separated and an appropriate amount was dissolved in pre-cooled saline to prepare a homogenate containing 10% liver tissue. The homogenate was centrifuged and supernatant obtained. MDA content and SOD activity was measured with xanthine oxidase and thiobarbituric acid (TBA) condensation methods, respectively, as per the kit manufacturer’s instructions.

### 3.13. Detecting the Expression Levels of PTP1B in Liver by Western Blot

Rat liver tissue was isolated, then frozen at −80 °C refrigerator and cell lysates were added. Protein concentration was determined by using BCA Protein Assay Kit (Thermo Scientific, Waltham, MA, USA). After electrophoresis, 60 mg proteins were transferred to polyvinylidene difluoride (PVDF) membranes. PTP1B antibody was added to the samples and then frozen overnight at 4 °C. The following day, the membranes were probed with a secondary antibody for 2 h. The bands were detected using enhanced chemiluminescence reagents. Protein bands were visualized by autoradiography and the intensities were analyzed by Image J (National Institutes of Health, v2.1.4.7, Bethesda, MD, USA, 2011).

### 3.14. Statistical Analysis

All data analyses were performed with SPSS 17.0 software (SPSS, version 17.0, New York, NY, USA, 2010). Data is expressed as mean ± standard deviation. Inter-group differences were assessed by *t*-test; *p* < 0.05 or *p* < 0.01 was considered indicative of a statistically significant difference.

### 3.15. Ethical Consideration

The study was approved by the Ethics Committee of School of Basic Medical Sciences, Jilin University, Jilin, China.

## 4. Conclusions

A water-soluble polysaccharide named CPOP was extracted from purslane. CPOP contains 48.3% total carbohydrates (by phenol-sulfuric acid), 10.3% proteins, 40.5% uronic acid, and is mainly composed of rhamnose, arabinose, xylose, mannose, glucose, and galactose in the ratio of 1:1.1:1.3:1.9:2.4:3.4. In this study, intragastric administration of CPOP was associated with a significant increase in the body weight and a significant improvement in glucose tolerance in the diabetic rats. CPOP appeared to significantly reduce FBG levels and elevate FINS and ISI levels in diabetic rats. In addition, CPOP appeared to significantly reduce TNF-α and IL-6 levels in diabetic rats, suggesting an anti-inflammatory effect. CPOP treatment was also associated with reduced MDA content and increased SOD activity in the liver tissue of diabetic rats, indicating its antioxidant properties. These results suggest that anti-diabetic effect of CPOP may be mediated via its antioxidant and anti-inflammatory effects.

## Figures and Tables

**Table 1:** Mean body weight of rats by study group ($\bar{x}$ ± SD, *N* = 8).

Group	Dose (mg/kg)	Baseline Mean Body Weight (g)	Mean Body Weight on Day 14 (g)	Mean Body Weight on Day 28 (g)
NC	–	208.31 ± 16.28	251.08 ± 26.21	299.23 ± 31.27
MC	–	209.48 ± 14.85	187.92 ± 15.17 **	150.42 ± 13.11 **
100-CPOP	100	207.75 ± 13.61	219.47 ± 22.68	247.36 ± 23.14
200-CPOP	200	209.62 ± 16.06	227.71 ± 24.15 ^#^	251.03 ± 22.47 ^##^
400-CPOP	400	208.83 ± 18.27	228.45 ± 19.21 ^##^	268.85 ± 25.98 ^##^
Glyburide	25	209.29 ± 16.22	236.25 ± 20.52 ^##^	275.21 ± 24.35 ^##^

** *p* < 0.01 vs. NC group (*N* = 8); ^#^ *p* < 0.05, ^##^ *p* < 0.01 vs. MC group (*N* = 8). Data expressed as mean ± standard deviation (SD), NC is the normal control group; MC is the model control group; 100-CPOP is the 100 mg/kg per body weight CPOP group; 200-CPOP (crude *Portulaca oleracea* L. polysaccharide) is the 200 mg/kg per body weight CPOP group; 400-CPOP is the 400 mg/kg per body weight CPOP group.

**Table 2:** Results of glucose tolerance test by study group ($\bar{x}$ ± SD, *N* = 8).

Group	Dose (mg/kg)	Mean Fasting Blood Sugar Level (mM)			
		0 min	30 min	60 min	120 min
NC	–	4.32 ± 0.88	13.65 ± 1.24	8.94 ± 1.47	4.89 ± 0.95
MC	–	16.04 ± 2.15 **	48.65 ± 3.29 **	42.32 ± 4.18 **	32.74 ± 2.82 **
100-CPOP	100	14.42 ± 2.07 ^#^	42.82 ± 2.81 ^##^	33.54 ± 3.23 ^##^	25.06 ± 2.67 ^##^
200-CPOP	200	12.36 ± 1.52 ^##^	33.98 ± 3.61 ^##^	24.77 ± 2.75 ^##^	18.91 ± 2.03 ^##^
400-CPOP	400	10.49 ± 1.84 ^##^	28.46 ± 3.05 ^##^	19.35 ± 2.36 ^##^	14.36 ± 1.42 ^##^
Glyburide	25	8.37 ± 1.26 ^##^	22.16 ± 2.19 ^##^	14.82 ± 1.63 ^##^	10.55 ± 1.34 ^##^

** *p* < 0.01 vs. NC group (*N* = 8); ^#^ *p* < 0.05, ^##^ *p* < 0.01 vs. MC group (*N* = 8). Data expressed as mean ± standard deviation (SD), NC is the normal control group; MC is the model control group; 100-CPOP is the 100 mg/kg per body weight CPOP group; 200-CPOP is the 200 mg/kg per body weight CPOP group; 400-CPOP is the 400 mg/kg per body weight CPOP group.

## References

[1] Westerhaus B., Gosmanov A.R., Umpierrez G.E. (2011). Diabetes prevention: Can insulin secretagogues do the job?. Prim. Care Diabetes.

[2] Defronzo R.A. (2009). From the triumvirate to the ominous octet: A new paradigm for the treatment of type 2 diabetes mellitus. Diabetes.

[3] Wild S., Roglic G., Green A., Sicree R., King H. (2004). Global prevalence of diabetes: Estimates for the year 2000 and projections for 2030. Diabetes Care.

[4] Kitabchi A.E., Temprosa M., Knowler W.C. (2005). Role of insulin secretion and sensitivity in the evolution of type 2 diabetes in the diabetes prevention program: Effects of life style intervention and metformin. Diabetes.

[5] Inzucchi S.E. (2002). Oral antihyperglycemic therapy for type 2 diabetes: Scientific review. JAMA.

[6] Xiang A.H., Peters R.K., Kjos S.L., Goico J., Ochoa C., Marroquin A., Tan S., Hodis H.N., Azen S.P., Buchanan T.A. (2004). Pharmacological treatment of insulin resistance at two different stages in the evolution of type 2 diabetes: Impact on glucose tolerance and β-cell function. J. Clin. Endocrinol. Metab..

[7] Mann J.F., Schmieder R.E., McQueen M., Dyal L., Schumacher H., Pogue J., Wang X., Maggioni A., Budaj A., Chaithiraphan S. (2008). Renal outcomes with telmisartan, ramipril, or both, in people at high vascular risk (the ONTARGET study): A multicentre, randomised, double-blind, controlled trial. Lancet.

[8] Nissen S.E., Wolsk I.K. (2007). Effect of rosiglitazone on the risk of myocardial infarction and death from cardiovascular causes. N. Engl. J. Med..

[9] Scarpello J.H., Hodgson E., Howlett H.C. (1998). Effect of metformin on bile salt circulation and intestinal motility in type 2 diabetes mellitus. Diabet. Med..

[10] Owen M.R., Doran E., Halestrap A.P. (2000). Evidence that metformin exerts its anti-diabetic effects through inhibition of complex 1 of the mitochondrial respiratory chain. Biochem. J..

[11] Zhen Z.Y., Wei L., Feng Z. (2015). Deciphering the therapeutic mechanisms of Xiao-Ke-An in treatment of type 2 diabetes in mice by a Fangjiomics approach. Acta Pharmacol. Sin..

[12] Li W.L., Zheng H.C., Bukuru J., de Kimpe N. (2004). Natural medicines used in the traditional Chinese medical system for therapy of diabetes mellitus. J. Ethnopharmacol..

[13] Liu J.P., Zhang M., Wang W.Y., Grimsgaard S. (2004). Chinese herbal medicines for type 2 diabetes mellitus. Cochrane Database Syst. Rev..

[14] Xie W., Xing D., Sun H., Wang W., Ding Y., Du L. (2005). The effects of *Ananas comosus* L. leaves on diabetic-dyslipidemic rats induced by alloxan and a high-fat/high-cholesterol diet. Am. J. Chin. Med..

[15] Xie W., Zhang Y., Wang N., Zhou H., Du L., Ma X., Shi X., Cai G. (2008). Novel effects of macrostemonoside A, a compound from Allium macrostemon Bung, on hyperglycemia, hyperlipidemia, and visceral obesity in high-fat diet-fed C57BL/6 mice. Eur. J. Pharmacol..

[16] Xie W.D., Zhao Y.N., Du L.J., Cai G.P. (2011). Scorpion in combination with Gypsum: Novel antidiabetic activities in streptozotocin-induced diabetic mice by up-regulating pancreatic PPAR*γ* and PDX-1 expressions. Evid. Based Complement. Altern. Med..

[17] Chauhan B.S., Johnson D.E. (2009). Seed germination ecology of *Portulaca oleracea* L.: An important weed of rice and upland crops. Ann. Appl. Biol..

[18] Lee J., Chauhan B.S., Johnson D.E. (2011). Germination of fresh horse purslane *(Trianthema portulacastrum)* seeds in response to different environmental factors. Weed Sci..

[19] D’Andrea R.M., Andreo C.S., Lara M.V. (2014). Deciphering the mechanisms involved in *Portulaca oleracea* (C4) response to drought: Metabolic changes including crassulacean acid-like metabolism induction and reversal upon re-watering. Physiol. Plant..

[20] Uddin M.K., Juraimi A.S., Hossain M.S. (2014). Purslane weed (*Portulaca oleracea*): A prospective plant source of nutrition, omega-3 fatty acid, and antioxidant attributes. Sci. World J..

[21] Zhao R., Zhang T., Zhao H. (2015). Effects of *Portulaca oleracea* L. polysaccharides on phenotypic and functional maturation of murine bone marrow derived dendritic cells. Nutr. Cancer.

[22] Zhou Y.X., Xin H.L., Rahman K., Wang S.-J., Peng C., Zhang H. (2015). *Portulaca oleracea* L.: A review of phytochemistry and pharmacological effects. BioMed Res. Int..

[23] Meng Y., Ying Z., Xiang Z., Hao D., Zhang W., Zheng Y., Gao Y., Ying X. (2016). The anti-inflammation and pharmacokinetics of a novel alkaloid from *Portulaca oleracea* L.. J. Pharm. Pharmacol..

[24] Rahdari P., Hosseini S.M., Tavakoli S. (2012). The studying effect of drought stress on germination, proline, sugar, lipid, protein and chlorophyll content in purslane (*Portulaca oleracea* L.) leaves. J. Med. Plants Res..

[25] Lee A.S., Lee Y.J., Lee S.M., Yoon J.J., Kim J.S., Kang D.G., Lee H.S. (2012). *Portulaca oleracea* ameliorates diabetic vascular inflammation and endothelial dysfunction in *db*/*db* mice. Evid. Based Complement. Altern. Med..

[26] Gong F., Li F., Zhang L., Li J., Zhang Z., Wang G. (2009). Hypoglycemic effects of crude polysaccharide from *Purslane*. Int. J. Mol. Sci..

[27] El-Sayed M.I.K. (2011). Effects of *Portulaca oleracea* L. seeds in treatment of type-2 diabetes mellitus patients as adjunctive and alternative therapy. J. Ethnopharmacol..

[28] Winzell M.S., Ahren B. (2004). The high-fat diet-fed mouse: A model for studying mechanisms and treatment of impaired glucose tolerance and type 2 diabetes. Diabetes.

[29] Leinonen E., Hurt-Camejo E., Wiklund O., Hultén L.M., Hiukka A., Taskinen M.R. (2003). Insulin resistance and adiposity correlate with acute-phase reaction and soluble cell adhesion molecules in type 2 diabetes. Atherosclerosis.

[30] Spranger J., Kroke A., Mohlig M., Hoffmann K., Bergmann M.M., Ristow M., Boeing H., Pfeiffer A.F. (2003). Inflammatory cytokines and the risk to develop type 2 diabetes. Diabetes.

[31] Ruan H., Hacohen N., Golub T.R., van Parijs L., Lodish H.F. (2002). Tumor necrosis factor-α suppresses adipocytes-specific genes and activates expression of preadipocyte genes in 3T3-L1 adipocytes: Nuclear factor-κB activation by TNF-α is obligatory. Diabetes.

[32] Naguib G., Al-Mashat H., Desta T., Graves D.T. (2004). Diabetes prolongs the inflammatory response to a bacterial stimulus through cytokine dysregulation. J. Investig. Dermatol..

[33] Lee H.B., Yu M.R., Yang Y.Q. (2003). Reactive oxygen species-regulated signaling pathways in diabetic nephropathy. J. Am. Soc. Nephrol..

[34] Susztak K., Raff A.C., Schiffer M. (2006). Glucose-induced reactive oxygen species cause apoptosis of podocytes and podocyte depletion at the onset of diabetic nephropathy. Diabetes.

[35] Koren S., Fantus I.G. (2007). Inhibition of the protein tyrosine phosphatase PTP1B: Potential therapy for obesity, insulin resistance and type-2 diabetes mellitus. Best Pract. Res. Clin. Endocrinol. Metab..

[36] Wang N., Zhang D., Mao X., Zou F., Jin H., Ouyang J. (2009). Astragalus polysaccharides decreased the expression of PTP1B through relieving ER stress induced activation of ATF6 in a rat model of type 2 diabetes. Mol. Cell. Endocrinol..

[37] Tamrakar A.K., Maurya C.K., Rai A.K. (2014). PTP1B inhibitors for type 2 diabetes treatment: A patent review (2011–2014). Expert Opin. Ther. Pat..

[38] Pan D., Wang L., Hu B., Zhou P. (2014). Structural characterization and bioactivity evaluation of an acidic proteoglycan extract from *Ganoderma lucidum* fruiting bodies for PTP1B inhibition and anti-diabetes. Biopolymers.

[39] Davis J.A., Sharma S., Mittra S., Sujatha S., Kanaujia A., Shukla G., Katiyar C., Lakshmi B.S., Bansal V.S., Bhatnagar P.K. (2012). Antihyperglycemic effect of *Annona squamosa* hexane extract in type 2 diabetes animal model: PTP1B inhibition, a possible mechanism of action?. Indian J. Pharmacol..

[40] Sun Y.X., Wang S.S., Li T.B., Li X., Jiao L., Zhang L. (2008). Purification, structure and immunobiological activity of a new water-soluble polysaccharide from the mycelium of *Polyporus albicans* (Imaz.) teng. Bioresour. Technol..

[41] Dubois M., Gilles K.A., Hamilton J.K., Gilles K.A., Hamilton J.K., Rebers P.A., Smith F. (1956). Colorimetric method for determination of sugars and related substances. Anal. Chem..

[42] Lowry O.H., Farr A.L., Rosebrough N.J., Farr A.L., Randall R.J. (1951). Protein measurement with the Folin-Phenol reagents. J. Biol. Chem..

[43] Filisetti-Cozzi T.M., Carpita N.C. (1991). Measurement of uronic acids without interference from neutral sugars. Anal. Biochem..

[44] Lehrfeld J. (1985). Simultaneous gas-liquid chromatographic determination of aldonic acids and aldoses. Anal. Chem..

